# Comparing the topological rank of journals in Web of Science and Mendeley

**DOI:** 10.1016/j.heliyon.2019.e02089

**Published:** 2019-07-29

**Authors:** Yurij L. Katchanov, Yulia V. Markova, Natalia A. Shmatko

**Affiliations:** a Institute for Statistical Studies and Economics of Knowledge, National Research University Higher School of Economics, 20 Myasnitskaya Ulitsa, Moscow 101000, Russian Federation; b American Association for the Advancement of Science, 1200 New York Ave NW, 20005, Washington, DC, USA

**Keywords:** Information science, Biological journals, Ranking, Bibliometrics, Altmetrics, Wakeby distribution

## Abstract

Recently, there has been a surge of interest in new data emerged due to the rapid development of the information technologies in scholarly communication. Since the 2010s, altmetrics has become a common trend in scientometric research. However, researchers have not treated in much detail the question of the probability distributions underlying these new data. The principal objective of this study was to investigate one of the classic problems of scientometrics—the problem of citation and readership distributions. The study is based on the data obtained from two information systems: Web of Science and Mendeley. Here we based on the concept of the cumulative empirical distribution function to explore the differences and similarities between citations and readership counts of biological journals indexed in Web of Science and Mendeley. The basic idea was to determine, for any journal, a “size” (it is said to be the topological rank) of citation and readership empirical cumulative distributions, and then to compare distributions of the topological ranks of Web of Science and Mendeley. In order to verify our model, we employ it to the bibliometric and altmetric research of 305 biological journals indexed in Journal Citation Reports 2015. The findings show that both distributions of the topological rank of biological journals are statistically close to the Wakeby distribution. The findings presented in this study add to our understanding of information processes of the scholarly communication in the new digital environment.

## 1 Introduction

The widespread adoption of information technologies and social media by the academic community substantially changed scholarly communication. Nowadays, researchers and academics actively use not only general social networks as Twitter and Facebook, but also academic social sites as Mendeley, Researchgate, and Academia.edu. The development of information technologies has led to the emergence of digital footprints of scientific communication such as download rates, blog and microblog counts, readership counts, links to scholarly Web spaces and journal websites. These factors increased the visibility of scholarly communication and made it more open, transparent, and rapid. Earlier bibliometrics was forced to rely primarily on citation counts. Now it could change and enlarge available quantitative datasets, metrics, and tools by using new digital footprints of scientific communication. The advent of new data extracted from social media gave birth to the new research field in scientometrics, namely altmetrics [1].

A number of studies have focused on similarities and differences between traditional and altmetric scholarly data. Other works investigated demographic and gender characteristics of scholarly networks' users, the influence of social media on researchers' professional activities, use of the social Web by highly cited researchers, and so on (see, e.g., [2], [3], [4], [5], [6], [7]). New communication technologies have enabled research on the impact of scientific research beyond traditional scholarly journals. Blog posts, tweets, articles and links from Wikipedia, citations from books—all this digital content became a rich source of data for various bibliometric studies [8], [9], [10], [11], [12], [13], [14]. This has encouraged the creation of new indicators, metrics, and tools for different aspects of scholarly communication and research output [13], [15], [16], [17], [18], [19]. All this gave rise to the tectonic changes in scientometrics, which some researchers called the scientific revolution in scientometrics [20], [21].

Altmetric studies of academic social networks and social media have shown that they reflect different aspects of scientific impact [22], [23], [24], [25], [26], [27], [28], [29]. The comparison of these new information sources with traditional bibliometric databases Web of Science and Scopus demonstrated that Mendeley was one of the most promising sources of altmetrics [30], [31], [32], [33], [34]. Mendeley fixes the reading actions of profiled users. It has the best coverage of the research literature [35], [36] and a large user population. Several studies have revealed significant correlations between citations and Mendeley readership counts [25], [37], [38], [39]. Costas et al. compared Web of Science citations and the Mendeley readership to identify the differences and similarities between the two distributions [40]. D'Angelo and Di Russo examine the existence of similarities between citation patterns and patterns of Mendeley readership counts [41]. The significant advantage of Mendeley reader counts is that readership statistics appear more quickly than citations. Data from several studies suggest that Mendeley reader counts may possibly predict future citations [32], [42], [43], [44], [45], [46], [47]. However, the adoption of new data and sources provides not only new insights into scholarly communication but also new pressing problems for scientometrics (see, e.g., [41], [48], [49], [50]).

For scientometrics to develop as a discipline, one should systematically use new data about scholarly communication, brought about by the advancement of social media and social network services. To date one needs to gain a general understanding of the differences and similarities between statistical distributions of Web of Science and Mendeley. Therefore, we turn to a comparison of data on citation and readership retrieved from the two systems.

The primary aim of this paper is to provide a systematic comparison of two information systems: Web of Science and Mendeley. In order to compare the two information systems on a systematical basis, we need to understand the statistical regularities underlying our data. For this purpose, we turn to a comparison of statistical distributions, characterizing the totality of scientific journals. It means that the unit of comparison is a sample of journals. We compare how one sample of journals is mapped into two different information systems. We would like to emphasize that the unit of comparison is a sample of journals and not its elements. The scientometric analysis of samples allows us to concentrate on the comparison of information systems instead of individual journals. Since we now have no ambition of developing a detailed theory of scholarly journals, obtaining the ranking order may indeed be the justified scientometric result.

In this paper, we refrain from direct tasks of evaluation and prediction of impact, prestige, or popularity of scholarly journals, and focus on studying statistic regularities. Without ranking of scholarly journals, scientometrics would be very different from the structure we know today. Here partial ordering is a mathematical way to give scholarly journals a structure. We stress that we do not use nor do we suggest the use of the concept of topological rank in evaluative scientometrics. Topological rank does not operationalize the influence or status of a scientific journal. The concept was created solely for comparison of the statistical laws that govern the citation distribution in Web of Science and the readership distribution in Mendeley.

The objective of the paper is to develop a ranking of biological journals based on a topological approach to the analysis of scientometric data. The paper also addresses the study of distributions of topological rank based on Web of Science and Mendeley. Our hypothesis is that scholars' attention in Web of Science is distributed similar to that of Mendeley.

## 2 Materials & Methods

The research data were drawn from three information sources: The Journal Citation Reports 2015 (an annual publication of journal rankings provided by Clarivate Analytics), the Web of Science Core Collection (a scientific citation indexing service maintained by Clarivate Analytics), and Mendeley (a reference manager and academic social network owned by Elsevier). The Journal Citation Reports 2015 was used to define the sample of journals under study. Data on journal citations were extracted from the Web of Science Core Collection. Information on readership counts was retrieved from the Mendeley reference manager.

The initial sample counted 429 journals listed in the category “Biology and biochemistry” of the Journal Citation Reports. For statistical reasons, we excluded journals, published less than 100 papers in two years. The constrained sample consisted of 305 journals. The impact factor of the Journal Citation Reports is calculated on the base of the dataset which implies a two-year publication window, a one-year citation window, and “article” and “review” document types. Following this logic, we extracted data on citation in the 2015 year for all “articles” and “reviews” published in the sample journals in 2013 and 2014 years. In the next step, using DOI (digital object identifier) of the papers, we collected information on Mendeley readership counts via the Mendeley Applications Programming Interface using R programming language. The resulting dataset was subjected to further statistical analysis.

## 3 Background

The starting point of our bibliometric investigation is obtained values of journal citation and readership counts. Since probability properties are to be expressed in terms of distribution functions, the connections between empirical data and statistical analysis are based on the concept of an empirical cumulative distribution function of citations/readerships, hereafter abbreviated as ECDF and denoted by $\overset{ˆ}{F}(x)$ (see Eq. (6) in Appendix 1). The ECDF is calculated as the fraction of citations/readerships smaller or equal to *x* (for more details, see [51, p. 5]). The journal citation/readership is characterized by ECDF, which contains all the statistical information it is possible to obtain about the journal. ECDF is sufficiently general and sufficiently convenient to be useful as a mathematical representation of the descriptive statistical information about a scholarly journal. In a sense, a journal *ξ* has an ECDF ${\overset{ˆ}{F}}_{\xi_{n}}(x)$ as its mathematical image, where *x* is the number of citations or readerships received by a paper (see Appendix 1 for more details). The journal *ξ* can be identified with the corresponding ECDF ${\overset{ˆ}{F}}_{\xi_{n}}(x)$.

This approach gives an easily understood scientometric picture in that it postulates a single fundamental object (namely, ECDF) to explain a variety of different observed statistical facts.

In the real world, scientific journals are the main communication medium. The scientometric understanding of this distributed medium is based largely on a concept of partial order ≼ (less than– or equal to–) relation (see Appendix 1). The notion of partial order relation appears to be a powerful means when evaluation and comparative study of scientific journals are required. Interpretation of partial order relation is essential for understanding what scientometrics tells us about scholarly communication, and usually, this interpretation means putting scientometric data into a sequence of ranks [52]. Systems of ranking journals arose out of our need to describe the intrinsic order of the scholarly communication. Almost all researchers accept the current order structure of scientific journals [53], [54], [55]. This recognized order, or journal ranking, directs not only the process of articles' submission but also scientists' reading and citing strategies [56]. An order structure ≼, in Bourbaki's sense [57], endows a totality of scholarly journals with defined scientometric meaning (e.g., “less impact/popularity/authority/… than or equal to”). It shows us how scientific journals group together to form a ranking. Order is such a basic concept in the scientometric explanation of science that it cannot be overemphasized.

Another important concept is entropy. It is a quantitative measurement of uncertainty associated with journals. In order to define entropy precisely, it is necessary to have a realistic but general enough mathematical model for journals. Such a model is given by the ECDF. In this paper, the random entropy *H* is given by

(1) $$\left(\sum_{i=0}^{m}k_{i}=n\right):H({\overset{ˆ}{F}}_{\xi_{n}})=-\ln\left(\frac{n!}{k_{0}!\ldots k_{m}!m^{n}}\right).$$

Here *n* denotes the number of papers of a scholarly journal, and $k_{i}$ is the number of papers which have been cited a total of *i* time. Details of how this is done have been explained in [58].

## 4 Theory

Let us denote by $S=\{\xi_{1},\xi_{2},\ldots ,\xi_{k}\}$ the sample from a population of scientific journals (see Appendix 1). Let *ξ* and *η* be journals from the sample *S*. We say that the set $C_{S}(\xi )=\{\eta \in S:\xi ≼\eta \}$ is a cone of *ξ* over *S*. We can look at this in another way: If $\eta \in C_{S}(\xi )$, then *ξ* precedes or equal to *η* (i.e., ξ≼η). Here, each journal ξ∈S has the smallest (with respect to inclusion) cone $C_{S}(\xi )$. The topology of *S* is generated by the set of all cones.

Now we want to define the nonstrict partial order ≼ on underlying sample of journals *S* in a natural and intuitive way. Before considering the problem, it will be useful to recall from [59, p. 204–205] the construction of a partially ordered set: Equipping the sample *S* with the some partial order is equivalent to direct specifying the “positive” cone $C_{S}^{+}\subset S$, with the following properties: $C_{S}^{+}+C_{S}^{+}\subset C_{S}^{+}$, $\alpha C_{S}^{+}\subset C_{S}^{+}$ (∀α≥0), $C_{S}^{+}\cap -C_{S}^{+}=\{0\}(\text{here}-{C_{S}}^{+}=\{-\xi :\xi \in C_{S}^{+}\})$. In other words, the sample *S* carries a relation, “the journal *ξ* precedes or equal to the journal *η*”. This relation on *S* is defined as follows: ξ≼η if $\eta -\xi \in C_{S}^{+}$. The nonstrict partial order ≼ on *S* and the positive cone $C_{S}^{+}$ are immediately connected: $\xi ≼\eta \Leftrightarrow \eta -\xi \in C_{S}^{+}$. In this way the use of the nonstrict partial order in this paper starts with the realization that the appropriate positive cone $C_{S}^{+}$ determines some natural order of scientific journals.

It might seem reasonable to propose a “gauge” of the smallest cone $C_{S}(\xi )$ by using the very properties of ${\overset{ˆ}{F}}_{\xi_{n}}(x)$. Determined in this way, it would give an idea of the positive cone $C_{S}^{+}$. We shall use the definition of $C_{S}^{+}$: If $\eta -\xi \in C_{S}^{+}$, then ${\overset{ˆ}{F}}_{\xi_{n}}(x)\leq {\overset{ˆ}{F}}_{\eta_{m}}(x)$. Let us introduce the function $f({\overset{ˆ}{F}}_{\xi_{n}})$ as follows:

(2) $$f({\overset{ˆ}{F}}_{\xi_{n}})=\frac{{\overset{ˆ}{F}}_{\xi_{n}}(x)}{1+{\overset{ˆ}{F}}_{\xi_{n}}(x)}.$$

Then: if ${\overset{ˆ}{F}}_{\xi_{n}}(x)\leq {\overset{ˆ}{F}}_{\eta_{m}}(x)$, then $f({\overset{ˆ}{F}}_{\xi_{n}})\leq f({\overset{ˆ}{F}}_{\eta_{m}})$. Obviously, the inequality

$$\int \frac{{\overset{ˆ}{F}}_{\xi_{n}}(x)}{1+{\overset{ˆ}{F}}_{\xi_{n}}(x)}\leq \int \frac{{\overset{ˆ}{F}}_{\eta_{m}}(x)}{1+{\overset{ˆ}{F}}_{\eta_{m}}(x)}$$

holds by the order preservation property of the operation of integration [60, p. 121].

Let a=min⁡{x} and b=max⁡{x} be integer positive numbers. Now we introduce the following concept. For every ECDF ${\overset{ˆ}{F}}_{\xi_{n}}(x)$ from *S* we set

(3) $$r(\xi )=\int_{a}^{b}\frac{{\overset{ˆ}{F}}_{\xi_{n}}(x)}{1+{\overset{ˆ}{F}}_{\xi_{n}}(x)}\mu (dx).$$

We shall say that the quantity r(ξ) is the *topological rank* of the journal *ξ* on *S*. The positive cone $C_{S}^{+}$ is determined with the help of the following relation: we consider $\eta -\xi \in C_{S}^{+}$ if r(η)−r(ξ)≥0. It now follows that if r(η)≤r(ξ), then η≼ξ.

**Figure 1:**
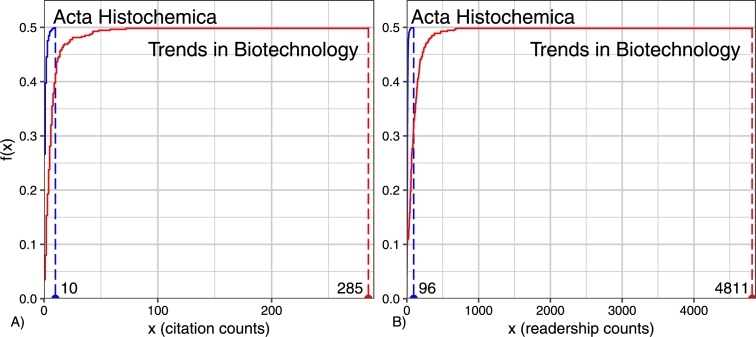
The positive real number which we assign as the area of the function $f(x)={\overset{ˆ}{F}}_{\xi_{n}}(x){\left(1+{\overset{ˆ}{F}}_{\xi_{n}}(x)\right)}^{-1}$ will be called the topological rank *r*(*ξ*) of the scientific journal *ξ*. It is easily seen that *r*(Trends in Biotechnology)[WoS] >*r*(Acta Histochemica)[WoS] and *r*(Trends in Biotechnology)[Mend] >*r*(Acta Histochemica)[Mend].

The definition of the topological rank will become more transparent to intuition if we note that the Lebesgue–Stieltjes integral [60, p. 152] in Eq. (3) can be treated as the area formed by the normalized ECDF $f({\overset{ˆ}{F}}_{\xi_{n}})$ given by Eq. (2). The area under the function $f({\overset{ˆ}{F}}_{\xi_{n}})$ gives information about the scale of the journal. The graphics of $f({\overset{ˆ}{F}}_{\xi_{n}})$ are ordered by inclusion (see Fig. 1). Metaphorically speaking, the topological rank r(⋅) is equal to the size of the (normalized) ECDF.

The nonstrict partial order ≼ on *S* defines journals as interrelated parts of the whole. All we can speak of are the relative positions of journals in the sample *S*. Here we adopt the topological rank r(⋅) as a tool to study the totality of scientific journals. The reader's attention is drawn to the fact that mathematicians use the term topological rank to denote the topological inclusion [61, p. 112].

Ultimately, a topological ranking for *S* is a mapping r(⋅) of the journals into the set of real positive numbers $R_{+}$ such that (∀ξ,η∈S):r(ξ)≤r(η)⇔ξ≼η. We can rearrange the scientific journals of *S* in ascending order $\xi_{\left(1\right)}≼\xi_{\left(2\right)}≼\cdots ≼\xi_{\left(k\right)}$. In this way, we make an assumption about the ordering of scientific journals with respect to ECDFs. In summary, we may say that the nonstrict partial order ≼ is expressed as the topological rank r(⋅), and the topological rank r(⋅) can be measured by the size of the normalized ECDF (see Eq. (3)).

Scientometrics today is not a strictly formalized system of laws, but rather a “methodological complex” that manifests itself in different ways depending on the form of research, scientific policy or management. It is important to draw a distinction between scientometrics as a form of research activity, and the outputs of that activity. More precisely, the topological rank is an axiomatically determined notion. An axiomatic foundation separates, in a sense, the mathematical aspect of the problem from all the rest. We do not need to explain how and where the concept of topological rank comes from. The topological rank simply becomes a primitive one, and its properties being described by mathematical definition. Clearly, the problem of how the topological rank thus introduced can be understood as a representation of real-world bibliometric phenomena remains open. But this problem is mostly removed by the remarkable fact that the bibliometric interpretation of the notion of topological rank is trivial since the scientometric theory is decidedly not complex and close to good sense (e.g., see [53]). Bibliometrics is a conceptual framework for understanding the statistical properties of scholarly communication [62], [63]. However, many of its essential elements can actually be described simply.

## 5 Results

In our first approximation, to achieve the goal of comparing the statistical laws that govern the citation distribution in Web of Science and the readership distribution in Mendeley, we use the two-sample Kolmogorov – Smirnov test to check whether ${\overset{ˆ}{F}}_{\xi_{n}}(x)$ and ${\overset{ˆ}{F}}_{\eta_{m}}(x)$ originate from the same distribution.

In our notation (9) (see Appendix 1) for the two-sample Kolmogorov – Smirnov test statistic, we study five variables:

1. The two-sample Kolmogorov – Smirnov test statistic $z(\xi ,\xi^{\prime })$ for all journals *ξ* in the sample *S*, to test whether ${\overset{ˆ}{F}}_{\xi_{n}}(x)$ from Web of Science and ${\overset{ˆ}{F}}_{\xi_{m}}(x)$ from Mendeley have the same distribution.
2. The two-sample Kolmogorov – Smirnov test statistic z(ξ,η)[WoS] for all possible pairs of citations' ECDFs (Web of Science), to check whether the two ECDFs are drawn from the same continuous distribution (see Fig. 2 A).

**Figure 2:**
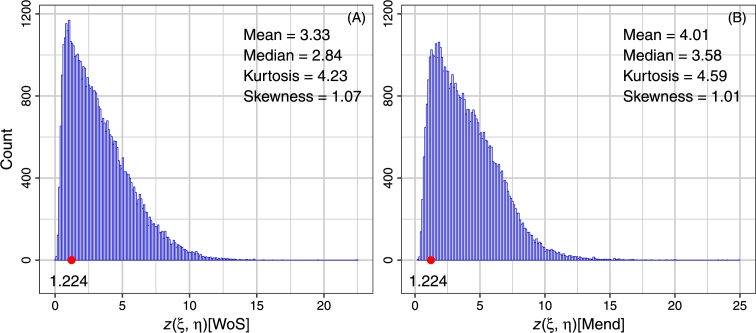
Histogram of the two-sample Kolmogorov – Smirnov test statistic *z*(*ξ*,*η*)[WoS] and *z*(*ξ*,*η*)[Mend].
3. The two-sample Kolmogorov – Smirnov test statistic z(ξ,η)[Mend] for all possible pairs of readerships' ECDFs (Mendeley), to check whether the two ECDFs are drawn from the same continuous distribution (see Fig. 2 B).
4. The eccentricity e(ξ)[WoS] in the Web of Science of a journal *ξ*, which is defined as the number e(ξ)[WoS]=max⁡z(ξ,η)[WoS].
5. The eccentricity e(ξ)[Mend] in the Mendeley of a journal *ξ*, which is defined as the number e(ξ)[Mend]=max⁡z(ξ,η)[Mend].

Let the significance level *α* be equal to 0.1; then z(α) be equal to 1.224 (see [51, p. 279–281]).

None of journals satisfies the condition $z(\xi ,\xi^{\prime })<1.224$. It can be said that the probability distributions of citation and readership of biological journals do not coincide. The inequality z(ξ,η)[WoS]>1.224 is valid for 80.89% of the values (see Fig. 2 A); in turn, the inequality z(ξ,η)[Mend]>1.224 holds for 90.67% of the values (see Fig. 2 B). The variable z(ξ,η)[WoS] is statistically related to the variable z(ξ,η)[Mend] (the Pearson correlation coefficient is equal to 0.638). This can be seen as indirect evidence that the citation distribution and readership distribution of biological journals are analogous.

**Figure 3:**
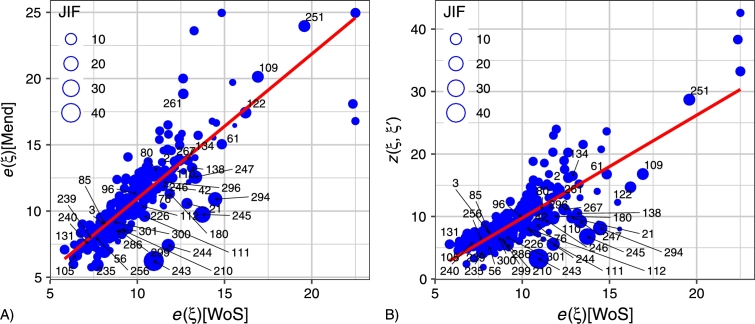
Scatterplot of the eccentricity in the Web of Science *e*(*ξ*)[WoS] against the eccentricity in the Mendeley *e*(*ξ*)[Mend] and the eccentricity in the Web of Science *e*(*ξ*)[WoS] against the two-sample Kolmogorov – Smirnov test statistic *z*(*ξ*,*ξ*′). Items having Journal Impact Factor equal to or higher than 5 are marked by numbers. List of journals see in Appendix 2.

There are statistical relationships between the eccentricity e(ξ)[WoS] in Web of Science and the eccentricity e(ξ)[Mend] in Mendeley on the one hand, and the eccentricity e(ξ)[WoS] in Web of Science and the variable $z(\xi ,\xi^{\prime })$ on the other, as shown by the following equations of linear regression (see Fig. 3):

1. $z(\xi ,\xi^{\prime })=-6.682+1.645\cdot e(\xi )\left[\mathrm{WoS}\right]\;(R^{2}=0.669)$;
2. $e(\xi )\left[\mathrm{Mend}\right]=0.027+1.091\cdot e(\xi )\left[\mathrm{WoS}\right]\;(R^{2}=0.726)$.

This result suggests that to a certain degree the structure of journals in Web of Science determined by the quantity z(⋅,⋅) is like the corresponding structure of journals in Mendeley. Furthermore, the list of top-10 journals having the maximum of the eccentricity e(ξ)[WoS] and the maximum of the eccentricity e(ξ)[Mend] overlaps significantly. The journals *Biochemical and Biophysical Research Communications*, *Bioresource Technology*, *Current Biology*, *eLife*, *International Journal of Clinical and Experimental Medicine*, *Journal of Biological Chemistry*, *Nucleic Acids Research* have the maximum distances z(⋅,⋅) to other journals in both systems.

In Appendix 2, we present the topological ranking for the 305 biological journals indexed in Web of Science and Mendeley based on the above procedure. Comparing the order of journals presented in Appendix 2, it is easy to see that two rankings are similar. For example, the list of top-five journals in Web of Science topological ranking contains *Nucleic Acids Research*, *Trends in Biotechnology*, *Nature Biotechnology*, *Nature Methods*, and *Science Translational Medicine*. In comparison, the top-five journals of Mendeley ranking are *Trends in Biotechnology*, *Nature Biotechnology*, *Nucleic Acids Research*, *Applied and Environmental Microbiology*, and *eLife*.

**Figure 4:**
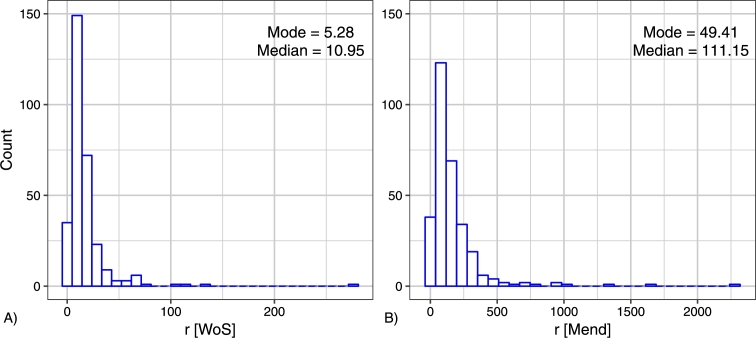
Histogram of the topological rank *r* for Web of Science and for Mendeley.

The mean *r* in Web of Science is 17.122 and in Mendeley is 166.14 (see the histogram in Fig. 4). It is not surprising since Mendeley being an independent information system has its own specific characteristics. For the distribution of *r*[WoS] and *r*[Mend], skewness is equal to 6.590 and to 5.334, respectively. The kurtosis of *r*[WoS] is equal to 64.304, and the one of *r*[Mend] is equal to 39.7. Thus, statistical analysis shows that the distribution of topological ranking in Web of Science is approximately symmetric and sharp as those of Mendeley. We can say that the topological ranking in Mendeley is more homogeneous than those of Web of Science (see also Table 3).

The order structure arises out of the basic idea that it is possible to compare two journals. In scientometrics, the concept of the order has become universal since the order structure almost everywhere precedes the introduction of other structures. However, another fundamental idea exists about closeness. The metric structure of scholarly journals is linked to this idea. In this context, one of the major results of our study can be formalized into an empirical model that introduces the partial order ⪯ on *S* discussed above such that it is *ε*-compatible with the uniform distance (7) (see in Appendix 1; for more details, see [64, p. 15–17]): (∀ξ,α,β,η∈S):ξ⪯α⪯β⪯η implies d(ξ,η)+2ε≥d(α,β). This inequality is an attempt directly to inscribe the distances d(ξ,η), d(α,β) between scientific journals ξ,α,β,η into the familiar framework of the ranking problem r(ξ)≤r(α)≤r(β)≤r(η). In our case *ε* for Web of Science dataset is equal 0.26 and for Mendeley dataset is equal 0.37. Thus, the ranking of scientific journals (3) is a mere expression of the metric structure. We say that the ordering of scholarly journals can be viewed as the result of the metric structure. In other words, if we know distances between scholarly journals, then we approximately know the journals' ranking. Seen from this angle, we are better able to understand the problem of ranking journals.

We used Spearman's rank correlation coefficient [65, p. 394–395] to assess statistical relationships between *r*, *z*, *H*, and Journal Impact Factor (see Table 1). As can be concluded from Table 1, there are significant positive correlations between *H*[WoS] and *H*[Mend], and between *r*[WoS], *r*[Mend] and z(ξ,ξ). This indicates the stochastic nature of the topological rank and distinctions between Web of Science and Mendeley. It is important to note that *H*[WoS] correlates strongly with *H*[Mend]. This evidence suggests that Web of Science and Mendeley display the same organizing principle localized at an entropy level.

**Table 1:** Spearman's rank correlation matrix (all correlations are significant at the 0.01 level, 2-tailed).

	JIF	*r*[WoS]	*r*[Mend]	*H*[WoS]	*H*[Mend]	*z*(*ξ*,*ξ*′)
JIF	1					
*r*[WoS]	0.834	1				
*r*[Mend]	0.685	0.738	1			
*H*[WoS]	0.987	0.833	0.700	1		
*H*[Mend]	0.791	0.713	0.771	0.813	1	
*z*(*ξ*,*ξ*′)	0.710	0.786	0.654	0.748	0.691	1

The topological rank r(ξ) is a nontrivial function of ${\overset{ˆ}{F}}_{\xi_{n}}(x)$. We can realize the intricate properties of *r*. Let us remember that the logarithm of *r* can be named the “magnitude” of *r*, that is the logarithm of entropy is the “magnitude” of entropy, etc. On the figures, we plot:

1. the magnitude of *r* in Mendeley versus the magnitude of *r* in Web of Science (Fig. 5A);

**Figure 5:**
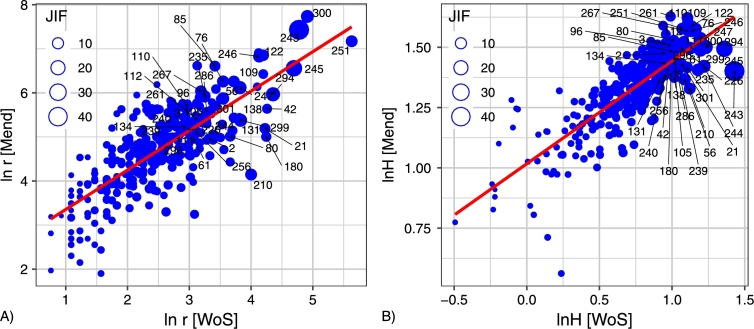
Scatterplot of the magnitude of topological rank *r* for Web of Science against the magnitude of topological rank *r* for Mendeley and the magnitude of entropy *H* for Web of Science against the magnitude of entropy *H* for Mendeley. Items having Journal Impact Factor equal to or higher than 5 are marked by numbers. List of journals see in Appendix 2.
2. the magnitude of *H* in Mendeley versus the magnitude of *H* in Web of Science (Fig. 5B);
3. the magnitude of *H* in Web of Science versus the magnitude of *r* in Web of Science (Fig. 6A);

**Figure 6:**
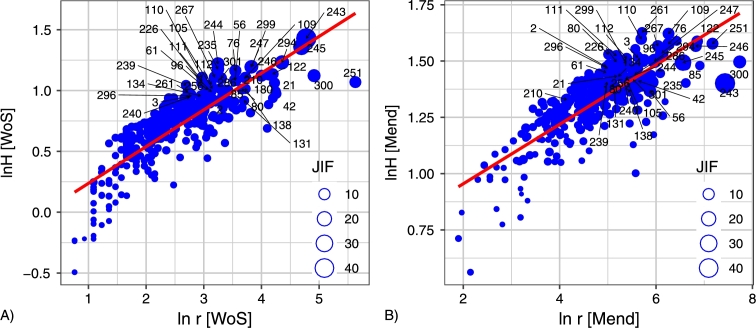
Scatterplot of the magnitude of topological rank *r* against the magnitude of entropy *H* for Web of Science and the magnitude of topological rank *r* against the magnitude of entropy *H* for Mendeley. Items having Journal Impact Factor equal to or higher than 5 are marked by numbers. List of journals see in Appendix 2.
4. the magnitude of *H* in Mendeley versus the magnitude of *r* in Mendeley (Fig. 6B).

We obtain the following equations of linear regression for four pairs of comparing quantities:

1. $\ln r\left[\mathrm{Mend}\right]=2.459+0.895\cdot \ln r\left[\mathrm{WoS}\right]\;(R^{2}=0.617)$;
2. $\ln H\left[\mathrm{Mend}\right]=1.017+0.426\cdot \ln H\left[\mathrm{WoS}\right]\;(R^{2}=0.665)$;
3. $\ln r\left[\mathrm{WoS}\right]=0.969+2.203\cdot \ln H\left[\mathrm{WoS}\right]\;(R^{2}=0.743)$;
4. $\ln r\left[\mathrm{Mend}\right]=0.691+0.132\cdot \ln H\left[\mathrm{Mend}\right]\;(R^{2}=0.617)$.

Clearly, the straight line y=a+bx on the double logarithmic plot implies that quantity *u* such that ln⁡u=y can be approximately expressed in the form $u\propto v^{b}$. Here ln⁡v=x, and *b* is the slope of the straight line y=a+bx.

Fig. 5 and Fig. 6 demonstrate a tendency toward a power law $u\propto v^{c}$ [66]. Hence, we can assume they are all a manifestation of a common regularity which can be formulated as follows: the main features of the citation distribution in Web of Science and readership distribution in Mendeley depend more on journals with high topological rank than on journals with low topological rank, although the latter are more numerous.

Today's scientometrician sees his/her task to explain the phenomena in science as complete if he or she can formulate the regularities of the sampled data in the form of probability distributions of the relevant quantities [62], [63]. In this paper, we focus on the Wakeby distribution [67, p. 44–46].

The Wakeby distribution of the topological rank *r* can be written as

(4) $$r=\zeta +\frac{\alpha }{\beta }(1-{\left(1-U\right)}^{\beta })-\frac{\gamma }{\delta }(1-{\left(1-U\right)}^{-\delta }).$$

Here *U* is a standard uniform random variable, and *β*, *γ*, *δ* are the continuous parameters, which are called shape parameters in statistics; *ζ* is the continuous location parameter, and *α* is the continuous scale parameter. The Eq. (4) determines the distribution, which is called the Wakeby distribution. The left and right ends of the Wakeby distribution, i.e. high and low frequencies, are related to those of a beta distribution and, respectively, a generalized Pareto distribution. Thereby, Eq. (4) states that, for δ>0, the probability distribution of *r* above *ζ* is a heavy-tailed distribution, i.e., explanate the origin of unequal probabilities. Furthermore, Eq. (4) allow us to propose what we call the statistical interpretation of *r*.

The obtained values are reported in Table 2. Applying goodness-of-fit test based on Kolmogorov–Smirnov's statistic, we demonstrate that the Wakeby distribution offers an acceptable level of accuracy. From this, it may be inferred that the Wakeby distribution describes well both the Web of Science and Mendeley topological ranks. Consequently, the Wakeby distribution can be useful in estimating *r* of a journal in Web of Science and Mendeley.

**Table 2:** Goodness of Fit—Summary.

Quantity	Kolmogorov–Smirnov test (α=0.1, d(α)=0.0703)	
	Distribution	Statistic
*r*[WoS]		
	Wakeby	0.0308
	Gen. Pareto	0.0581
	Lognormal (3P)	0.0404
	Gen. Extreme Value	0.0383

*r*[Mend]		
	Wakeby	0.0297
	Gen. Pareto	0.0717
	Lognormal (3P)	0.0506
	Gen. Extreme Value	0.0375

Table 3 lists the parameters of the Wakeby distribution for *r*[WoS] and *r*[Mend].

**Table 3:** Parameters of the Wakeby distribution.

Quantity	Parameters				
	*α*	*β*	*γ*	*δ*	*ζ*
*r*[WoS]	10.289	1.307	5.253	0.480	2.563
*r*[Mend]	142.44	1.89	57.719	0.457	10.541

## 6 Discussion

We do not state that quantities r[WoS] and r[Mend] have the same distribution. We believe that topological ranks in Web of Science and Mendeley indicate the same organizing principle. We empirically establish this principle as the Wakeby distribution, which is a statistical law of r[WoS] and r[Mend]. Here the parameters of the Wakeby distribution of r[WoS] and r[Mend] are not identical (see Table 3).

When we construct a topological rank using Web of Science citations and Mendeley readerships for the same sample of journals, in reality, we study one and the same information structure that manifests itself in two different forms.

The citation and readership are two different facets of one social process that could be called the “memory of science” [68], [69]. They are both part of scientific practices; nevertheless, they have some important differences. In a fact, the citation is a more selective process; the scientists and researchers read more papers than they cite (cf. [39], [44], [49]). Hence, a paper has less chance to be cited in Web of Science than to have readers in Mendeley. The readership indicates attention and interest of a reader in the topic of the paper, while the citation shows not only the interest of the author but also evaluation and a peer review of the paper's content. The references are prerequisites for the production of new scientific knowledge, but we cannot say the same about papers that have been read. It can be assumed that the citation mainly presents the contribution and scientific impact of a journal, while the readership reveals the societal impact of a journal and its popularity (cf. [70]). Finally, the citation and readership have different social meaning both to researchers and journals. The citation is a stake of the struggle for scientific recognition between researchers or between journals. The citation provides the basis for different metrics for journal ranking [55]. These metrics determine publication and scientific policy, influence scientific management, and researchers' attention. On the contrary, Mendeley readership data presently have no such social meaning [71] and are free from direct practical consequences for researchers or journals. Thus, this complex combination of similarities and differences shapes the information processes under study that we describe with the topological rank.

The topological rank r(⋅) proposed in this paper is, nevertheless, of a phenomenological kind and does not claim to be the scientometric theory. A general approach of the present study consists in topological rank r(⋅) being rather the way of describing bibliometric phenomena than the primary fact. The advantage of the topological rank r(⋅) is in the lack of assumptions in regard to the structure of the citation/readership process, along with the absence of groundless hypotheses about the citation/readership practices.

Let us address a heuristic question: What kind of organizing principle would give the Wakeby distribution of *r*? In the interest of mathematical simplicity, we omit the details that are not essential. Instead of dealing with Eq. (4), we opt for the simplest expression:

(5) $$r\propto \kappa_{1}U^{\beta }+\;\kappa_{2}U^{-\delta }.$$

If we stare at Eq. (5) thoroughly and long enough we see that if we use the change of variables r(U)⟼ln⁡w(U), then $w\propto \kappa_{1}\exp(\beta U)+\kappa_{2}\exp(-\delta U)$: So that *r* can be formally treated as the superposition of two colliding “waves of probability” in steady state. The first one (the term in $\kappa_{1}$) corresponds to an “incident” wave, propagating from left to right. The second one (the term in $\kappa_{2}$) corresponds to a “reflected” wave, propagating from right to left. We may metaphorically express it as follows: the “influence” runs forward and the “reflection” runs backward. Notice that the “probabilistic waves” in Eq. (5) have only heuristic meaning.

## 7 Conclusions

The paper shows that biological journals in Web of Science and Mendeley demonstrate the same Wakeby distribution of topological rank (cf. [25], [41], [72]). This finding suggests that biological journals in Web of Science and Mendeley are subjected to an analogous ranking, being influenced by a socially similar communication environment and selection mechanism. Common offline processes of scientific activity structure researchers' attention and produce the isomorphic ranking of biological journals in different information systems. When we rank journals, we seek correspondences between social relations in the field of science and relations in the totality of journals. Hence, it is not surprising that the distribution of topological rank in Web of Science is similar to the distribution of topological rank in Mendeley.

The results of the analysis suggest that Mendeley demonstrated statistical regularities not so unlike those found in Web of Science. In broad terms, information processes displayed in Mendeley are isomorphic to the ones exhibited by Web of Science. It is highly likely that distribution of the societal impact of journals resembles scientific impact distribution. Isomorphism of the two information processes supports the opinion that study of the readership data in Mendeley, available before citation data, may aid with prediction of journals citation in Web of Science. We hypothesize that at the core of information processes studied by bibliometrics as well as altmetrics are the same social regularities of the field of science. Figuratively speaking, Web of Science and Mendeley correspond to each other like two authorized translations of the same poem into different languages: They are not word-by-word identical, but isomorphous. Such result can motivate further scientometric works to uncover relationships between Web of Science and Mendeley.

Authors and editors of scientific journals tend to believe that readership/citation is determined by the special qualities of the articles. While it may be somewhat true, one should bear in mind that readership/citation is a social action [73]. A social action, in its turn, cannot be determined only by the object's characteristics [71]. More important are the characteristics of the subject. The statistics of readership/citation is determined by specific characteristics of the readership/citation subjects. Moreover, one should consider the social context of the actions (see, inter alia, [68], [74], [75]). It follows that the citation statistics in Web of Science resembles the readership statistics in Mendeley to the same extent that the readership of Web of Science mirrors that of Mendeley.

In conclusion, the limitations of the presented study should be made explicit. Actually, all the above analysis was performed on the dataset limited to papers published in biological journals in 2013–2014 years and indexed in the Web of Science Core Collection. These limitations give directions for our further research work. We think it would be practical and useful to make some comparison studies. Especially it would be interesting to compare physics journals with journals in computer science and in engineering. The second perspective option is a comparative study of social services important for scientists and researchers such as ResearchGate, Academic.edu or Twitter.

## Declarations

### Author contribution statement

Yurij Katchanov, Yulia Markova, Natalia Shmatko: Conceived and designed the experiments; Performed the experiments; Analyzed and interpreted the data; Contributed reagents, materials, analysis tools or data; Wrote the paper.

### Funding statement

The article was prepared within the framework of the Basic Research Program at the National Research University Higher School of Economics (HSE) and supported within the framework of a subsidy by the Russian Academic Excellence Project “5-100”.

### Competing interest statement

The authors declare no conflict of interest.

### Additional information

Supplementary content related to this article has been published online at https://doi.org/10.1016/j.heliyon.2019.e02089.

No additional information is available for this paper.

## Supplementary material

The following Supplementary material is associated with this article:

APPENDIX.PDF Appendix 1 includes background information on the notion of partial order and the empirical cumulative distribution function. Appendix 2 contains the list of biological journals under study with the main indicators. APPENDIX.PDF
