# Supplementary material for: Comparing the topological rank of journals in Web of Science and Mendeley
Source: Heliyon. 2019 Jul 29;5(7):e02089. doi: 10.1016/j.heliyon.2019.e02089 (PMC6667838; doi:10.1016/j.heliyon.2019.e02089)
Supplement: APPENDIX.PDF — Appendix 1 includes background information on the notion of partial order and the empirical cumulative distribution function. Appendix 2 contains the list of biological journals under study with the main indicators. [file mmc1.pdf]

Appendix 1. Partial order and empirical cumulative distribution function

The statistical representation of scientific journals involves complex multidimensional data. Usually, researchers tend to reduce complexity, making scientometric information emerge in the form of indicators or rankings of scholarly journals. The notion of nonstrict partial order is designed precisely to deal with this kind of problems [76], being a mathematical construction solely based on an algebraic relation, here on the concept of the binary relation $\preccurlyeq$ that is reflexive $(\xi \preccurlyeq \xi)$, antisymmetric $\big((\xi \preccurlyeq \eta) \wedge (\eta \preccurlyeq \xi) \Rightarrow (\xi \sim \eta)$, where the symbol $\sim$ denotes an equivalence relation$\big)$, and transitive $\big((\xi \preccurlyeq \eta) \wedge (\eta \preccurlyeq \zeta) \Rightarrow (\xi \preccurlyeq \zeta)\big)$. For a full treatment of this subject, we refer the reader to [77]. Nonstrict partial order relation $\preccurlyeq$ induces a structure on the totality of scientific journals: If $\xi \preccurlyeq \eta$ holds, we say "η follows or equal to ξ" or "ξ precedes or equal to η".

The starting point of the present investigation is scientometric data in the form of a set of independent and identically distributed observations $X_1, \dots, X_n$ (Web of Science citation counts or Mendeley readership counts) from a distribution $F(x)$. An estimator of $F(x)$ is obtained by the ECDF $\hat{F}_{\xi_n}(x)$. In turn, $\hat{F}_{\xi_n}(x)$ of the vector of observations (also called a sample) $\xi_n = \{X_1, \dots, X_n\}$ is the function

$$\hat{F}_{\xi_n}(x) = \frac{1}{n} \sum_{i=1}^{n} I_{[X_i \le x]}, \tag{6}$$

where

$$I_{[X_i \le x]} = \begin{cases} 1, & X_i \le x, \\ 0, & X_i > x. \end{cases}$$

It is well known, see [51, p. 6–7], that the ECDF $\hat{F}_{\xi_n}(x)$ converges almost surely to the corresponding distribution function $F(x)$ as $n \to \infty$ by the Glivenko–Cantelli theorem. In addition, we assume that the scientometric state of a scientific journal ξ is mathematically represented by its ECDF $\hat{F}_{\xi}$.

In the space of all ECDFs, to every pair of journals ξ, η corresponds the uniform (or Kolmogorov) distance

$$d(\xi, \eta) = \sup_x \left\{ \left| \hat{F}_{\xi_n}(x) - \hat{F}_{\eta_m}(x) \right| \right\} \tag{7}$$

between them [64, p. 15–17], whose fundamental properties are completely described by means of a collection of axioms . The well-known Smirnov's theorem [65, p. 192] claims that

$$\lim_{n\to\infty,\, m\to\infty} \Pr\left(\sqrt{\frac{nm}{n+m}}d(\xi,\eta) \geq v\right) = 1 - 2\sum_{k=1}^{\infty}(-1)^k \exp\bigl(-2k^2v^2\bigr), \quad (8)$$

where $\Pr(\cdot)$ denotes a probability. It remains to note that the calculation of the significance level of the two-sample Kolmogorov – Smirnov test is based on the assertion (8). For the sake of brevity, we introduce the following notation:

$$z(\xi,\eta) = \sqrt{\frac{nm}{n+m}}d(\xi,\eta). \quad (9)$$

Let α be a significance level. Then the null hypothesis H_0 ($\hat{F}_{\xi_n}(x)$ and $\hat{F}_{\eta_m}(x)$ come from the same distribution) is rejected, if $z \geq z(\alpha)$. If we combine this with (8), we find a critical value $z(\alpha)$ from $\Pr\bigl(z \geq z(\alpha)\bigr) = \alpha$ [51, p. 281].

Appendix 2. Biological journals in the sample

No.	Journal title	r[WoS]	H[WoS]	r[Mend]	H[Mend]
1	3 BIOTECH	11.896	1.251	486.400	3.742
2	ACS CHEM BIOL	27.558	2.589	142.977	4.370
3	ACS SYNTH BIOL	15.690	2.594	277.401	4.553
4	ACTA BIOCH BIOPH SIN	8.580	1.914	100.702	3.534
5	ACTA BIOCHIM POL	7.162	1.462	141.552	3.572
6	ACTA DIABETOL	17.589	2.181	47.030	3.743
7	ACTA HISTOCHEM	5.278	1.613	46.080	3.231
8	ACTA PHYSIOL	10.000	2.358	110.677	3.830
9	ADV NUTR	16.165	2.640	246.526	4.239
10	ALGAL RES	11.896	2.553	278.351	4.335
11	AM J PHYSIOL-ENDOC M	10.948	2.418	122.552	4.029
12	AM J PHYSIOL-REG I	10.474	2.256	97.377	3.851
13	AM J RESP CELL MOL	14.267	2.372	257.451	3.770
14	AM J TRANSL RES	12.370	2.264	276.926	3.604
15	AMINO ACIDS	15.216	2.257	74.578	3.818
16	ANAL BIOCHEM	22.336	1.983	102.602	3.901
17	ANAT REC	5.748	1.694	97.377	3.860
18	ANN ENDOCRINOL-PARIS	8.580	1.317	103.077	3.331
19	ANN ANAT	6.218	1.545	49.405	3.484
20	ANN HUM BIOL	13.318	1.569	39.431	3.666
21	ANTIOXID REDOX SIGN	68.403	2.895	179.551	4.191

22	APPL ENVIRON MICROB	61.754	2.423	1000.825	4.393
23	APPL BIOCHEM BIOTECH	7.634	1.716	136.802	3.684
24	APPL BIOCHEM MICRO+	3.879	1.049	24.235	2.238
25	APPL IMMUNOHISTO M M	8.107	1.654	51.304	2.937
26	APPL MICROBIOL BIOT	20.437	2.303	172.901	4.333
27	ARCH BIOCHEM BIOPHYS	14.267	2.169	180.501	3.985
28	ARCH BIOL SCI	2.969	0.803	28.033	2.410
29	ARTIF CELL NANOMED B	2.969	1.280	23.760	2.870
30	BIOANALYSIS	10.474	2.054	113.052	3.491
31	BIOCHEM BIOPH RES CO	15.216	2.060	328.226	3.421
32	BIOCHEM ENG J	18.538	2.071	167.201	4.170
33	BIOCHEM J	35.157	2.319	111.152	4.008
34	BIOCHEM SOC T	19.962	2.143	126.827	4.118
35	BIOCHEM SYST ECOL	4.343	1.381	57.479	3.387
36	BIOCHEMISTRY-US	23.760	2.204	297.826	4.015
37	BIOCHEMISTRY-MOSCOW+	6.690	1.590	79.328	3.571
38	BIOCHEM CELL BIOL	5.278	1.573	27.084	3.184
39	BIOCHEM MOL BIOL EDU	3.420	0.900	51.304	3.601
40	BBA-BIOENERGETICS	19.487	2.622	184.301	4.229
41	BBA-BIOMEMBRANES	18.063	2.426	316.826	4.171
42	BBA-GEN SUBJECTS	70.778	2.648	281.201	4.101
43	BBA-MOL CELL BIOL L	20.437	2.625	191.426	4.206
44	BBA-PROTEINS PROTEOM	17.114	2.241	118.752	4.051
45	BIOCHIMIE	12.844	2.231	129.677	4.054
46	BIODEGRADATION	5.278	1.924	35.632	3.563
47	BIOELECTROCHEMISTRY	8.580	2.246	38.006	3.729
48	BIOELECTROMAGNETICS	5.278	1.766	36.581	3.433
49	BIOENGINEERED	3.879	1.761	50.830	3.438
50	BIOESSAYS	15.216	2.601	146.777	4.237
51	BIOFACTORS	14.741	2.500	199.026	3.786
52	BIOFUEL BIOPROD BIOR	11.896	2.453	84.553	3.958
53	BIOLOGIA	3.420	1.152	38.006	3.158
54	BIOL CHEM	9.526	2.120	176.701	3.623
55	BIOL RES	8.107	1.580	56.529	3.657
56	BIOL REV	34.682	2.965	354.826	3.925
57	BIOL RHYTHM RES	3.420	1.145	43.230	3.162
58	BIOL TRACE ELEM RES	6.690	1.830	49.405	3.427
59	BIOL BULL+	2.138	0.788	24.235	2.542
60	BIOL OPEN	11.896	1.936	88.828	3.998
61	BIOMACROMOLECULES	21.386	2.740	142.502	4.259
62	BIOMED RES INT	28.983	1.766	325.376	4.232
63	BIOMED OPT EXPRESS	16.639	2.396	94.527	4.081
64	BIOMETALS	11.896	1.893	43.230	3.496
65	BIOMOL NMR ASSIGN	2.969	1.076	14.741	2.795
66	BIOORG CHEM	5.278	1.977	55.104	3.412

67	BIOPHYS J	18.063	2.342	179.076	4.489
68	BIOPOLYMERS	12.370	1.985	137.752	3.666
69	BIORESOURCE TECHNOL	28.033	2.604	262.201	4.728
70	BIOSCIENCE	10.474	2.431	258.876	4.213
71	BIOSCI BIOTECH BIOCH	6.218	1.467	55.579	3.263
72	BIOSYSTEMS	5.278	1.730	52.254	3.606
73	BIOTECH HISTOCHEM	3.420	1.432	14.267	2.964
74	BIOTECHNIQUES	8.580	1.820	125.402	3.787
75	BIOTECHNOL BIOTEC EQ	2.969	0.825	44.180	3.006
76	BIOTECHNOL ADV	34.682	3.201	522.025	4.836
77	BIOTECHNOL APPL BIOC	4.343	1.439	55.104	3.222
78	BIOTECHNOL BIOENG	20.437	2.498	151.052	4.423
79	BIOTECHNOL BIOPROC E	6.218	1.515	60.329	3.342
80	BIOTECHNOL BIOFUELS	38.956	2.752	150.577	4.616
81	BIOTECHNOL J	10.000	2.337	100.227	4.236
82	BIOTECHNOL LETT	9.053	1.694	108.302	3.700
83	BIOTECHNOL PROGR	6.690	1.923	278.351	3.960
84	BMB REP	11.422	2.154	96.902	3.513
85	BMC BIOL	30.408	2.727	739.575	4.405
86	BMC BIOTECHNOL	6.690	2.045	112.102	4.137
87	BMC SYST BIOL	8.107	1.991	231.326	4.338
88	BRAZ ARCH BIOL TECHN	3.879	0.930	93.577	3.477
89	BRAZ J BIOL	2.969	0.940	38.956	3.466
90	CALCIFIED TISSUE INT	26.609	2.165	137.277	3.730
91	CELL BIOSCI	9.053	2.168	164.351	3.829
92	CELL BIOCHEM BIOPHYS	17.589	1.704	97.852	3.358
93	CENT EUR J BIOL	3.879	1.299	35.632	3.170
94	CHEMBIOCHEM	13.318	2.164	91.203	3.879
95	CHEM BIOL DRUG DES	60.329	1.992	463.125	3.516
96	CHEM BIOL	22.810	2.694	316.826	4.517
97	CHEM PHYS LIPIDS	10.948	2.126	283.101	3.805
98	CHRONOBIOL INT	12.844	2.350	86.453	4.108
99	CLIN LIPIDOL	5.278	1.145	86.453	2.879
100	CSH PERSPECT MED	17.589	2.407	294.976	4.529
101	COLLOID SURFACE B	16.639	2.447	138.702	4.064
102	COMP BIOCHEM PHYS A	7.634	1.878	62.704	3.728
103	COMP BIOCHEM PHYS B	4.809	1.670	46.555	3.522
104	COMP BIOCHEM PHYS C	6.218	2.018	31.357	3.626
105	COMPR PHYSIOL	23.285	2.768	223.726	4.075
106	CR BIOL	5.278	1.463	44.655	3.581
107	CRYOBIOLOGY	6.690	1.814	80.753	3.751
108	CRYOLETTERS	4.343	1.232	12.844	2.646
109	CURR BIOL	41.331	3.022	522.500	5.061
110	CURR OPIN BIOTECH	19.487	3.025	296.401	4.943
111	CURR OPIN CHEM BIOL	19.487	2.945	176.701	4.589

112	CURR OPIN STRUC BIOL	22.810	2.861	218.976	4.537
113	CURR PROTEIN PEPT SC	5.748	2.018	85.978	3.456
114	CYTOM PART A	13.318	2.190	132.527	4.053
115	CYTOTECHNOLOGY	5.278	1.591	51.779	3.536
116	CYTOTHERAPY	45.130	2.265	230.376	3.980
117	DATABASE-OXFORD	9.053	1.938	119.227	4.067
118	DIABETES METAB	11.422	2.251	79.803	3.937
119	DIS MODEL MECH	26.609	2.478	200.926	4.282
120	DOKL BIOCHEM BIOPHYS	2.138	0.793	7.162	2.287
121	ELECTRON J BIOTECHN	3.420	1.387	93.102	3.577
122	ELIFE	66.503	3.114	946.675	4.892
123	ENDOCRINE	10.948	2.189	87.403	3.644
124	ENDOCR J	9.526	1.889	64.129	3.135
125	ENDOCRINOLOGY	24.710	2.479	205.201	3.752
126	ENG LIFE SCI	17.114	1.851	83.603	3.845
127	ENZYME MICROB TECH	8.580	2.098	68.878	3.935
128	EUR BIOPHYS J BIOPHY	6.218	1.564	42.280	3.337
129	EUR J ENDOCRINOL	19.013	2.438	232.751	3.910
130	EXP CLIN ENDOCR DIAB	7.634	1.691	48.930	3.379
131	EXP MOL MED	40.381	2.502	171.476	3.921
132	EXP PHYSIOL	10.000	2.158	108.302	3.958
133	EXPERT REV PROTEOMIC	10.948	2.162	84.553	3.574
134	FASEB J	19.013	2.605	185.251	4.186
135	FEBS J	30.883	2.508	439.851	4.245
136	FEBS LETT	33.257	2.396	350.551	4.205
137	FEBS OPEN BIO	8.580	1.903	66.503	3.725
138	FREE RADICAL BIO MED	41.331	2.759	229.426	3.950
139	FREE RADICAL RES	12.844	2.205	112.577	3.444
140	FRONT BIOSCI-LANDMRK	10.948	2.053	50.355	3.287
141	FRONT PHYSIOL	21.861	2.458	215.651	4.539
142	GEN COMP ENDOCR	9.053	2.127	130.152	3.922
143	GEN PHYSIOL BIOPHYS	3.420	1.268	8.580	1.754
144	GLYCOBIOLOGY	9.053	2.224	84.553	3.738
145	GLYCOCONJUGATE J	9.526	1.826	69.353	3.477
146	HEMOGLOBIN	2.969	1.228	32.782	2.961
147	HISTOCHEM CELL BIOL	19.962	2.038	112.577	3.776
148	HISTOL HISTOPATHOL	9.053	1.826	89.303	3.138
149	HISTOPATHOLOGY	19.962	2.247	98.327	3.541
150	HIST BIOL	5.278	1.655	42.755	2.868
151	HORM METAB RES	10.474	1.852	31.357	3.343
152	INDIAN J BIOCHEM BIO	8.107	1.356	100.702	3.007
153	INDIAN J EXP BIOL	6.218	1.514	41.806	3.311
154	INDIAN J PATHOL MICR	2.969	1.007	17.114	2.789
155	INTERFACE FOCUS	7.634	1.989	79.328	4.107
156	INT J BIOCHEM CELL B	29.458	2.445	138.227	3.795

157	INT J BIOL MACROMOL	17.114	2.247	154.376	3.538
158	INT J BIOL SCI	18.063	2.426	171.951	3.895
159	INT J BIOMATH	4.809	1.155	6.690	2.038
160	INT J CLIN EXP MED	8.107	1.503	67.453	3.093
161	INT J MORPHOL	2.533	0.803	24.710	2.483
162	INT J PEPT RES THER	3.879	1.265	49.405	2.866
163	INT J RADIAT BIOL	7.634	1.768	49.405	3.112
164	IUBMB LIFE	7.162	2.087	118.277	3.740
165	J ANAT	21.386	1.908	74.578	4.033
166	J APPL MICROBIOL	9.526	1.964	88.828	3.962
167	J APPL PHYSIOL	17.114	2.246	185.251	4.306
168	J BIOCHEM	23.760	1.920	113.527	3.632
169	J BIOENERG BIOMEMBR	5.748	1.792	35.157	3.406
170	J BIOL CHEM	29.933	2.510	156.276	4.138
171	J BIOL REG HOMEOS AG	4.809	1.482	11.422	2.822
172	J BIOMED NANOTECHNOL	13.793	2.400	27.084	3.327
173	J BIOMED SEMANT	7.162	1.766	63.654	3.656
174	J BIOMOL NMR	28.033	2.353	96.902	3.760
175	J BIOMOL STRUCT DYN	10.474	1.663	45.130	3.257
176	J BIOPHOTONICS	14.267	2.174	49.880	3.714
177	J BIOSCI BIOENG	10.000	1.878	274.551	4.062
178	J BIOSCIENCES	5.748	1.604	181.451	3.752
179	J BIOTECHNOL	26.134	2.132	257.451	4.336
180	J BONE MINER RES	69.828	2.699	149.152	4.021
181	J COMP PHYSIOL A	5.748	1.884	111.627	4.170
182	J COMPUT BIOL	21.386	1.559	53.204	3.265
183	J CYTOL	3.879	1.024	14.741	2.323
184	J ENDOCRINOL INVEST	9.526	1.840	79.803	3.370
185	J ENDOCRINOL	19.013	2.510	196.176	3.932
186	J ENZYM INHIB MED CH	21.861	1.941	25.659	2.894
187	J EVOL BIOCHEM PHYS+	2.138	0.611	16.639	2.169
188	J EXP BIOL	17.589	2.217	193.801	4.443
189	J GEN PHYSIOL	11.896	2.478	148.677	3.891
190	J HISTOCHEM CYTOCHEM	6.690	1.928	84.553	3.621
191	J IND MICROBIOL BIOT	10.000	2.156	141.552	4.054
192	J INHERIT METAB DIS	19.962	2.353	197.126	3.843
193	J INNOV OPT HEAL SCI	3.879	1.141	26.134	2.675
194	J INORG BIOCHEM	10.474	2.246	46.080	3.737
195	J LIPID RES	22.810	2.512	747.175	4.065
196	J MATH BIOL	6.218	1.693	41.331	3.517
197	J MICROSC-OXFORD	8.107	1.914	104.502	3.823
198	J MOL ENDOCRINOL	9.053	2.180	97.852	3.745
199	J MOL HISTOL	7.634	1.926	29.458	2.950
200	J MOL RECOGNIT	18.063	1.761	233.226	3.396
201	J MORPHOL	6.218	1.592	87.403	3.749

202	J NANOBIOTECHNOL	30.883	2.172	298.776	3.996
203	J NUTR BIOCHEM	23.760	2.526	364.326	4.214
204	J OVARIAN RES	7.634	2.088	94.527	3.502
205	J PEPT SCI	11.422	1.816	103.077	3.424
206	J PHOTOCH PHOTOBIO B	23.760	2.217	130.152	3.736
207	J PHYSIOL-LONDON	28.508	2.544	270.276	4.369
208	J PHYSIOL BIOCHEM	20.911	1.847	138.702	3.360
209	J PHYSIOL PHARMACOL	11.422	2.091	44.180	2.992
210	J PINEAL RES	54.629	3.071	63.179	3.776
211	J PROTEOME RES	21.861	2.474	195.701	4.174
212	J PROTEOMICS	13.318	2.433	161.501	4.349
213	J RADIAT RES	5.748	1.698	50.355	3.612
214	J STEROID BIOCHEM	16.639	2.385	184.301	3.816
215	J STRUCT BIOL	10.948	2.107	239.876	4.034
216	J THEOR BIOL	15.216	1.932	92.627	4.076
217	J TRACE ELEM MED BIO	7.634	1.986	96.427	3.395
218	LETT APPL MICROBIOL	5.748	1.678	79.328	3.686
219	LIFE SCI	12.370	2.158	191.901	3.863
220	LIPIDS	4.809	1.856	45.130	3.509
221	LIPIDS HEALTH DIS	7.634	1.950	124.927	3.856
222	LUMINESCENCE	5.748	1.556	22.336	2.881
223	MACROMOL BIOSCI	12.844	2.355	196.176	3.754
224	MATH BIOSCI	4.343	1.593	64.604	3.670
225	MATRIX BIOL	10.948	2.556	121.127	4.158
226	METAB ENG	19.487	2.896	176.701	4.467
227	METABOLISM	14.267	2.556	124.452	4.243
228	METABOLOMICS	28.508	2.195	250.326	3.995
229	METHODS	19.013	2.349	275.026	4.594
230	MICROB CELL FACT	10.000	2.376	311.601	4.457
231	MICRON	10.474	1.857	99.752	3.718
232	MICROSCOPY-JPN	7.162	1.529	122.077	3.492
233	MICROSC RES TECHNIQ	7.634	1.416	40.381	3.370
234	MOL CELL ENDOCRINOL	13.793	2.453	144.402	3.886
235	MOL ASPECTS MED	25.659	3.247	372.876	4.081
236	MOL BIOSYST	24.235	2.175	163.876	3.823
237	MOL BIOTECHNOL	14.741	1.798	339.151	3.718
238	MOL ENDOCRINOL	13.318	2.283	105.927	3.693
239	MOL METAB	14.741	2.846	144.877	4.080
240	MOL THER-NUCL ACIDS	15.690	2.578	216.126	3.983
241	MOL CELLS	17.114	2.077	98.327	3.797
242	MYCOBIOLOGY	4.343	1.083	69.353	3.120
243	NAT BIOTECHNOL	118.752	4.175	1685.775	4.060
244	NAT CHEM BIOL	25.659	3.387	214.226	4.142
245	NAT METHODS	109.252	3.897	704.900	4.453
246	NAT PROTOC	62.229	3.103	919.600	4.792

247	NAT STRUCT MOL BIOL	45.605	3.302	454.100	4.646
248	NEUROENDOCRINOL LETT	8.580	1.372	383.801	3.231
249	NEW BIOTECHNOL	9.526	2.214	161.026	4.183
250	NITRIC OXIDE-BIOL CH	20.437	2.329	96.427	3.745
251	NUCLEIC ACIDS RES	278.351	2.915	1300.550	4.841
252	NUCLEOS NUCLEOT NUCL	4.809	1.328	17.589	2.645
253	NUTR RES	9.526	2.086	97.852	4.154
254	OBESITY	33.257	2.366	146.302	4.343
255	OMICS	8.107	2.112	104.027	3.602
256	ONCOIMMUNOLOGY	22.336	2.600	167.676	4.006
257	OPEN BIOL	19.013	2.548	207.576	4.178
258	ORG BIOMOL CHEM	38.956	2.361	84.078	3.306
259	PEPTIDES	18.063	2.097	118.277	3.730
260	PFLUG ARCH EUR J PHY	10.000	2.262	120.177	3.981
261	PHILOS T R SOC B	15.216	2.690	302.101	5.093
262	PHOTOCH PHOTOBIO SCI	9.526	2.010	55.104	3.431
263	PHOTOCHEM PHOTOBIOL	6.218	1.834	41.806	3.434
264	PHYS BIOL	8.107	1.759	47.980	3.759
265	PHYSIOL MEAS	8.107	1.867	108.302	3.769
266	PHYSIOL RES	5.278	1.758	123.977	3.534
267	PLOS BIOL	24.235	3.037	418.951	4.791
268	PLOS COMPUT BIOL	66.503	2.555	617.975	4.902
269	PREP BIOCHEM BIOTECH	3.420	1.433	17.589	3.023
270	PRION	12.370	2.108	103.077	3.695
271	PROCESS BIOCHEM	19.487	2.039	316.351	4.056
272	PROG BIOPHYS MOL BIO	9.053	2.097	124.927	4.102
273	PROSTAG LEUKOTR ESS	9.053	2.208	64.129	3.494
274	PROTEIN CELL	12.844	2.338	99.277	4.040
275	PROTEIN PEPTIDE LETT	7.634	1.400	56.054	2.857
276	PROTEIN ENG DES SEL	9.053	2.012	122.552	3.938
277	PROTEIN EXPRES PURIF	7.634	1.569	146.302	3.676
278	PROTEIN J	6.218	1.397	251.276	3.091
279	PROTEIN SCI	23.285	2.191	232.751	4.085
280	PROTEINS	22.336	1.990	241.776	3.942
281	PROTEOME SCI	4.809	1.829	44.180	3.555
282	PROTEOMICS	22.336	2.440	159.126	4.131
283	PROTEOM CLIN APPL	10.948	2.119	67.928	3.757
284	RADIAT ENVIRON BIOPH	4.343	1.845	28.033	3.208
285	RADIAT RES	14.741	2.203	88.353	3.481
286	REDOX BIOL	26.609	2.761	259.826	4.224
287	REGUL PEPTIDES	5.278	1.762	24.710	3.242
288	REPROD BIOL ENDOCRIN	10.474	1.958	258.876	3.896
289	REV BIOL TROP	2.969	0.909	62.229	3.213
290	RNA	34.207	2.481	197.126	4.383
291	RNA BIOL	16.165	2.395	188.101	4.303

292	SAUDI J BIOL SCI	5.748	1.800	114.477	4.104
293	SCI CHINA LIFE SCI	5.278	1.863	57.004	3.491
294	SCI TRANSL MED	77.903	3.427	390.451	4.486
295	STEROIDS	15.690	2.068	85.978	3.619
296	STRUCTURE	16.165	2.603	147.252	4.340
297	THYROID	20.437	2.447	59.379	3.583
298	TRANSL CANCER RES	8.580	1.622	264.101	2.723
299	TRENDS BIOCHEM SCI	46.080	3.307	216.601	4.429
300	TRENDS BIOTECHNOL	135.852	3.074	2285.700	4.464
301	TRENDS ENDOCRIN MET	27.084	3.011	251.276	4.124
302	TURK J BIOL	4.343	1.439	20.911	3.144
303	ULTRASTRUCT PATHOL	2.969	1.203	12.844	2.681
304	WORLD J MICROB BIOT	5.748	1.696	114.477	3.791
305	Z NATURFORSCH C	2.969	1.185	10.000	2.647
